# Hydrolysable Tannin Supplementation Alters Digestibility and Utilization of Dietary Protein, Lipid, and Carbohydrate in Grass Carp (*Ctenopharyngodon idellus*)

**DOI:** 10.3389/fnut.2019.00183

**Published:** 2019-12-17

**Authors:** Jingting Yao, Peng Chen, Andrews Apraku, Gaigai Zhang, Zhongyuan Huang, Xueming Hua

**Affiliations:** ^1^Centre for Research on Environmental Ecology and Fish Nutrition (CREEFN) of the Ministry of Agriculture, Shanghai Ocean University, Shanghai, China; ^2^Key Laboratory of Freshwater Aquatic Genetic Resources, Ministry of Agriculture, Shanghai, China; ^3^National Demonstration Center for Experimental Fisheries Science Education, Shanghai Ocean University, Shanghai, China; ^4^Editorial Office, Journal of Shanghai Ocean University, Shanghai Ocean University, Shanghai, China

**Keywords:** *Ctenopharyngodon idellus*, hydrolysable tannin, metabolism, protein, lipid, carbohydrate

## Abstract

Tannin, an antinutritional component of plant proteins was fed to grass carp (*Ctenopharyngodon idellus*, 8. 18 ± 0.81 g) for 8 weeks to investigate their tolerance levels. Semi-purified diets (T0, T1, T2, and T3) with varying levels of hydrolysable tannin (0, 0.75, 1.25, and 1.75% respectively) were used. No significant difference was obtained in weight gain, while feed conversion ratio of T0 was significantly lower than T2. Muscle protein content of T0 and T3 were significantly higher than T2, while lipid content of T0 was significantly higher than other groups. Muscle and hepatic glycogen in T0 were significantly lower than other groups. Muscle saturated fatty acids in T3 were significantly higher than T0, and lowest in T1 and T2, while the poly-unsaturated fatty acids in T1 and T2 were higher than T0 and lowest in T3. Significant increases were obtained in trypsin and amylase activities as tannin levels increased, the lipase activity of T0 and T1 was significantly higher than T2 and T3. Activities of hepatic alanine aminotransferase and aspartate aminotransferase decreased with increasing tannin level. The total protein in serum of T2 was significantly higher than T0 and T1 and lowest in T3, whereas globulin of T2 was significantly higher than T0 and T3 and lowest in T1, while albumin of T1 was significantly higher than other groups. Urea nitrogen of T0 was significantly higher than other groups, triglyceride and total cholesterol significantly increased with tannin level and decreased in T3, significant decreases were obtained in low-density lipoprotein cholesterol and high-density lipoprotein cholesterol in T3. mRNA expression of intestinal TOR was significantly upregulated as dietary tannin increased. In hepatopancreas, the expression of glucokinase in T1 was significantly higher than T2, and lowest in T0 and T3, pyruvate kinase in T2 was significantly higher than T0 and T1 and lowest T3. The expression of lipoprotein lipase upregulated as tannin level and downregulated in T3, and fatty acid synthase downregulated as tannin level. In conclusion, grass carp could tolerate 1.75% dietary tannin without influencing growth. However, 1.25% tannin impaired digestion and metabolism of protein, decreased the deposition of lipid and promoted utilization of carbohydrate.

## Introduction

Fishmeal is an excellent macro-ingredient in aquatic feeds. However, the high cost, increasing demands and supply fluctuations of fishmeal have seriously restricted the sustainable development of aquaculture (1). Therefore, a steady supply and relatively nutrition-balanced products have considerably been expanded to meet the demand for aquatic feeds (2, 3).

Plant proteins such as rapeseed meal (RM) have been widely used as fishmeal substitutes in aquatic feeds (4, 5). However, high level of dietary RM induces poor growth performance, low nutrition digestibility, and stress response of fish due to its anti-nutritional factors (ANFs), such as phytic acid, glucosinolate, and tannin (6, 7). Liu et al. (8) reported that, 4.7 g/kg phytic acid decreased the apparent digestibility and utilization of amino acids and minerals of grass carp (*Ctenopharyngodon idellus*). Burel et al. (9) suggested that, 26 μmol/g glucosinolate disturb the thyroid hormone and decrease growth performance in rainbow trout (*Oncorhynchus mykiss*). However, limited information is available about the influence and underlying mechanism of dietary tannin on fish.

Tannins are polyphenolic substances with various chemical structures, which are generally divided into hydrolysable and condensed tannins (10). Tannins exist in plant feed ingredients widely. There are 52 mg/kg (wet matter) total tannin in Pomegranate (*Punica granatum* L.), 0.13–1.69 mg/g (DM) total tannin in broad bean, 6–9 mg/g (DM) total tannin in alfalfa cultivars, 1.2–5.3 mg/g (DM) total tannin in soybean meal, 0.7% (DM) hydrolysable tannin in dehulled rapeseed meal, and 1.2–3.7% total tannin in rapeseed meal (11–18). Both condensed tannin and hydrolysable tannin exist in RM. Condensed tannins are known to induce some physiological effects such as antioxidant, anti-hypertensive, as well as antimicrobial activities. Also, hydrolysable tannic could influence the expression of some biological activities including antimutagenic, anticancer, and antioxidant properties (19). Both hydrolysable and condensed tannins could bind with feed components like proteins, vitamin, and minerals to influence digestion (20). Previous study on rats showed that, tannin decreased the digestibility of protein and lipid, and the fermentability of undigestible protein was affected (21). Another study on rats suggested tannin decreased α-amylase activity as well as in digestibility of available carbohydrates (22). Ge et al. (23) demonstrated that persimmon tannin could effectively reduce the cellular cholesterol levels in both HepG2 and Caco-2 cells via inhibiting the cholesterol accumulation-related genes.

Hydrolysable tannins are widely explored in mammal animals. In a study of pigs, a linear decrease in feed conversion ratio and daily body weight gain was observed with increasing dietary tannic level (24). However, in the studies of ruminant, 71.3 g/kg total tannin improved the body weight gain and feed conversion ratio in Holstein bulls (25). This may be because, tannin-protein complexes are less soluble and less accessible to proteolytic enzymes in the ruminal pH, and thereby reduce the degradation of proteins in the rumen and increased the degradation of proteins in the intestine (26). These results revealed dosage-dependent and species-special effect of tannin. There are many biological differences between mammals and aquatic organisms, and few studies have been carried out to characterize the effect of tannin on aquatic animals. Therefore, the underlying mechanism will be of great significance for aquatic animals.

Prusty et al. (27) reported that, Indian major carp (*Labeo rohita*) could tolerate 2% dietary hydrolysable tannin, and Buyukcapar et al. (20) showed Nile Tilapia (*Oreochromis niloticus*) could tolerate 0.5% dietary hydrolysable tannin. These studies focus on omnivorous fish. Herbivorous fish have been noted to tolerate higher quantity of plant proteins and ANFs, and as such makes it the best choice in further investigations.

Grass carp is a typical herbivorous euryhaline fish that has been largely cultured in East Asia. Previous studies suggested that grass carp could tolerate 35% dietary RM, perhaps it is an excellent model with strong tolerance to tannin (28, 29).

The present study characterized the biological indices of grass carp fed diets with graded levels of hydrolysable tannin supplementation, aimed to evaluate its impact on growth and nutrition metabolism of grass carp, so as to better understand the fundamental mechanism of antinutritional effects induced by hydrolysable tannin and provide theoretical evidence in rapeseed meal utilization.

## Materials and Methods

### Experimental Fish and Environmental Conditions

Grass carp with an average initial weight of 8.18 ± 0.81 g were obtained from Huzhou Nanxun Honghao Fisheries and transported to Shanghai Ocean University culture facility located at Binhai, Shanghai, China.

Fish were randomly distributed to 16 indoor net cages with volumes of 2.5 cubic meters each and 70–80 cm water depth fixed in indoor tanks; 20 fish per cage, randomly divided into four groups with four replicates. The fish were fed with commercial diets for a one-week acclimatization period.

Environmental and water quality indicators were monitored daily over the course of the experiment. The tanks were supplied with filtered pond water and uninterrupted aeration to maintain an appropriate level of dissolved oxygen (>5 mg/L), ammonium nitrogen (<0.6 mg/L), and the water temperature varied 24–32°C.

### Diets and Feeding

The feeding trial lasted for 8 weeks and all fish were fed to apparent satiation three times daily (7:00 am, 12:00 pm, and 17:00 pm). Uneaten diet was collected 30 min after feeding, dried and weighed for correction of the feed intake. Four isonitrogenous (crude protein 31.01%) and isoenergetic (gross energy 17.34–17.63 MJ/kg) semi-purified diets were formulated. These diets contained 0% (T0), 0.75% (T1), 1.25% (T2), and 1.75% (T3) hydrolysable tannin supplementation, respectively, in which the major protein sources were casein and gelatin, and the major lipid source was soybean oil (Table 1).

**Table 1:** Composition and nutrient levels of diets (dry basis) %.

**Ingredients**	**T0**	**T1**	**T2**	**T3**
Casein	24.00	24.00	24.00	24.00
Gelatin	6.00	6.00	6.00	6.00
Wheat middling	35.00	35.00	35.00	35.00
Corn starch	17.50	17.50	17.50	17.50
Soybean oil	1.40	1.45	1.45	1.45
Ca(H_2_PO_4_)_2_·H_2_O	2.00	2.00	2.00	2.00
Choline chloride	1.00	1.00	1.00	1.00
Compound vitamins and minerals^a^	1.50	1.50	1.50	1.50
Sodium carboxymethylcellulose	11.60	10.8	10.30	9.8
Hydrolysable tannin^b^	0.00	0.75	1.25	1.75
Nutrient levels				
Crude protein	31.01	31.01	31.01	31.01
Crude fat	2.35	2.40	2.40	2.40
Crude ash	2.28	2.32	2.38	2.54
Cellulose	12.20	11.41	10.91	10.42
Gross energy/(MJ/kg)	17.63	17.51	17.42	17.34
Tannin	0.00	0.75	1.25	1.75

a *Compound vitamins and compound minerals were obtained from Hangzhou Haihuang Feed Development Co. Ltd*.

b *Hydrolysable tannin was bought from Wuhan Baixing Bio-Technique Co. Ltd., effective substance content is 99%*.

All ingredients were grounded into fine powder through a 60-mesh sieve, weighed according to the formula and thoroughly mixed with soybean oil, then water was added and pelleted with an experimental feed mill, dried for about 12 h in a ventilated oven at 40°C. After drying, the diets were broken and sieved into proper pellet size (1.5 mm in diameter), and well-stored at a cool dry well-ventilated place.

### Sample Collection

At the end of the 8-week feeding period, all fish in each cage were counted and weighed. Seven fish were randomly pooled from each cage (28 fish/group), anesthetized with eugenol solution (100 ppm) and killed via sharp blow to the cranium prior to dissection. After body weight and body length was measured, the exterior of the individuals was wiped clean and the abdomen was opened at the ventral mid line to separate and weigh hepatopancreas, intestine and viscera. Weight gain (WG), feed efficiency ratio (FER), feed intake (FI), relative intestine weight, relative hepatopancreas weight, and relative viscera weight were calculated by the following formulae:

$$\begin{array}{l} \text{Survival rate }(\%)=\text{final number of fish}\\ \;\times \text{ 100/initial number of fish}\\ \text{WG }=(\text{final weight }-\text{ initial weight})\\ \;\times \text{ 100/}(\text{initial weight})\\ \text{FCR}=\text{total feeding/}(\text{final weight}\\ \;-\text{initial weight + dead weight})\\ \text{FI}=\text{total feeding }\times \text{100/}[\text{feeding period}\\ \;\times (\text{final weight +initial weight})\text{/2}]\\ \text{Relative intestine weight }(\%)=\text{intestine mass}\times \text{100/body mass}\\ \text{Relative hepatopancreas weight }(\%)=\text{hepatopancreas mass}\\ \;\times \text{100/body mass}\\ \text{Relative viscera weight }(\%)=\text{viscera mass }\times \text{100/body mass} \end{array}$$

Blood samples from seven fish each cage (28 fish/ group) were collected from the caudal vein, centrifugated at 4,000 × g for 10 min at 4°C, to obtain the serum and stored at −20°C for further analysis. Intestine, hepatopancreas and muscle were weighed and collected from the selected fish, frozen on ice prior to storage at −20°C.

Following the feeding period, other three fish were sampled from each group randomly, anesthetized and killed as above. The intestine and hepatopancreas were collected from the selected fish. For RNA extraction, the tissues were fixed in 1.5 ml of RNAse-free centrifuge tubes and then stored at −80°C. The expression of TOR mRNA (target of rapamycin), GK mRNA (glucokinase), PK mRNA (pyruvate kinase), CTP1 mRNA (Carnitine palmitoyl transferase), FAS mRNA (fatty acid synthase), and LPL mRNA (lipoprotein lipase) in different tissues were determined.

### Muscle Composition Analysis

Muscle were dried at 70°C for 2 h and then to a constant weight at 105°C to determine the moisture content; protein was determined by measuring nitrogen (N × 6.25) using the Kjeldahl method, lipid by ether extraction using chloroform-methanol extraction (30) and crude ash by combustion at 550°C. Muscle glycogen were measured using specific analytical procedures and commercially obtained kits for fish (Jiancheng Bioengineering Institute, Nanjing, China). For amino acids (except for methionine), the muscle was freeze-dried, and then hydrolyzed with 6 N HCl at 110°C for 24 h, and analyzed by amino acid analyzer (Biochrom Ltd, Cambridge, Science Park, England). The lipid in muscle was esterified to fatty acid methyl esters (FAMEs) with 14% BF3 in methanol (31) and extracted with hexane, then fatty acids profile was detected in duplicated on an HP6890 gas chromatograph according to the method described by Huang et al. (32), the statistical analysis of fatty acid referring to the method of Li et al. (33).

### Digestive Enzymes Activities and Biochemical Parameters

Hepatopancreas and intestine samples were homogenized by adding sterile 0.85% saline solution to prepare 10% (W:V) homogenates, and then centrifuged at 4,000 × g for 10 min at 4°C. Supernatants were used to analyze the activities of digestive enzyme and biochemical parameters in 12 h.

Serum parameters like total protein (TP), albumin (ALB), total cholesterol (TC), triglyceride, low density lipoprotein cholesterol (LDL-C), high density lipoprotein cholesterol (HDL-C), and urea nitrogen (UN) and activities of alanine aminotransferase (ALT), aspartate aminotransferase (AST) and hepatic glycogen of hepatopancreas were measured using specific analytical procedures and commercially obtained kits (Jiancheng Bioengineering Institute, Nanjing, China).

The intestinal activity of trypsin was analyzed following the method of Natalia et al. (34). The activity of alpha-amylase was measured according to Worthington (35). The activity of lipase was assayed based on measurement of fatty acids released due to enzymatic hydrolysis of triglycerides in a stabilized emulsion of olive oil (36) using specific analytical procedures and commercially available kits (Jiancheng Bioengineering Institute, Nanjing, China). All enzyme assays were performed in quadruplicate.

### Isolation of Total RNA

Total RNA was extracted via the Trizol Reagent, based on the manufacturer's instructions (Invitrogen, USA). The RNA concentration was determined via absorbance at 260 nm with a UV spectrophotometer (Thermo Fisher Scientific, USA). We assessed the purity by measuring the RNA content and the OD260/OD280 ratio. The total RNA samples typically yielded 100 ng/μLRNA and an OD260/OD280 ratio between 1.8 and 2.0.

### Real-Time Quantitative Reverse Transcription PCR

Based on conserved gene cDNA sequences of β-actin, TOR, LPL, FAS, CPT1, GK, and PK of grass carp, fluorescence quantitative primers (Table 2) were designed and synthesized with Primer5 software by the Jiangsu Jin Weizhi company (Jiangsu City, P.R. China). Real-time fluorescence quantitative PCR were measured via ABI7500 according to instructions using the SuperReal SYBR Green (TianGen, FP205) of the two-step RT-PCRkit. The volume of the reactions was 20 μL, in which 10 μL Goldstar PCR Master Mix (2 ×), 0.6 μL Primer, 0.4 μL Rox, 2 μL CDNA, and 7 μL dH_2_O were used. A fluorescent signal was collected every 0.3°C from 60 to 95°C with β-actin chosen as reference gene. The thermocycling conditions were as follows: initiated with transcript reaction at 95°C for 15 min, followed by forty cycles at 95°C for 15 s and 60°C for 30 s. Melting curve analysis was performed over a range of 50–95°C to verify that a single PCR product was generated. Both the target gene and the internal control β-actin gene were amplified from each cDNA sample under identical conditions on the same plate. Each sample was amplified in triplicate. The relative mRNA expression of genes was calculated via the 2^−ΔΔCt^ method. We compared the relative mRNA expression levels of all experimental groups to those of the T0 group.

**Table 2:** The primers for qPCR.

**Gene name**	**Accession**	**Forward**	**Reverse**
TOR	JX854449	ATGCTGTGATCCCACTTTCC	GCATAATGCGGTGTTCAATG
LPL	FJ716100.1	TGTGATTGTGGTGGACTGGT	GATGCAGTTTCTCCCAAGGA
FAS	GQ466046	GCGCTGTCGAGTGTTTACAA	CCTTTGCCCTGAGTGTTGAG
CPT1	JF728839	ATCTGCCTGGACCTCAGAGA	TGGCCACAGACAGAGAGTTG
GK	GU065314.1	TTTGGATAAGGGCATTCTGC	GTGTCATTCACCATGGCAAC
PK	JQ951928.1	TCATGCTGTCTGGAGAAACG	CAGAGCTCAGAGGGGTCAAA
β-actin	DQ211096	AAGGCCAACAGGGAAAAGAT	CATCACCAGAGTCCATCACG

*TOR, Target of Rapamycin; LPL, lipoprotein lipase; FAS, fatty acid synthase; CPT1, Carnitine palmitoyl transferase; GK, glucokinase; PK, pyruvate kinase*.

### Data Analysis

Data were presented as means ± SD and analyzed via one-way analysis of variance (ANOVA) in SPSS version 17.0. Differences in mean values were analyzed via Duncan's multiple range test when ANOVA identified differences among groups. The level of significance was set to *P* < 0.05.

## Result

### Survival and Growth Performance

No significant (*P* > 0.05) difference was observed in survival rate, weight gain rate, relative hepatopancreas weight, and relative intestine weight (Table 3). The FCR and FI of T2 was higher than T1 and T3 (*P* > 0.05), and significantly higher than T0 (*P* < 0.05). The relative viscera weight of T0 was significantly (*P* < 0.05) higher than other groups.

**Table 3:** Survival and growth performance of grass carp (means ± SD, *n* = 4).

	**T0**	**T1**	**T2**	**T3**
Survival rate%	96.25 ± 4.79	95.00 ± 5.78	98.75 ± 2.50	97.50 ± 2.89
Weight gain %	230.96 ± 14.90	225.91 ± 25.79	228.01 ± 20.22	227.85 ± 19.84
Feed conversion ratio	1.87 ± 0.11^a^	1.94 ± 0.08^ab^	2.04 ± 0.07^b^	1.98 ± 0.11^ab^
Feed intake %	3.57 ± 0.14^a^	3.66 ± 0.11^ab^	3.84 ± 0.15^b^	3.75 ± 0.11^ab^
Relative intestine weight %	3.62 ± 0.50	3.60 ± 0.48	3.46 ± 0.38	3.59 ± 0.34
Relative hepatopancreas weight %	1.60 ± 0.08	1.63 ± 0.19	1.69 ± 0.04	1.69 ± 0.13
Relative viscera weight %	9.79 ± 0.31^b^	8.21 ± 0.28^a^	7.77 ± 0.40^a^	7.88 ± 0.17^a^

*In the same line, different lower-case letters indicate significant differences (P < 0.05); values with no letter mean no significant difference (P > 0.05)*.

### Muscle Composition

No significant (*P* > 0.05) difference was observed in muscle moisture and ash. The protein content of T0 and T3 were significantly (*P* < 0.05) higher than T2 and lowest in T1(Table 4). The muscle lipid content decreased significantly (*P* < 0.05) with tannin level. The content of muscle glycogen in T0 was significantly (*P* < 0.05) lower than other groups. No significant (*P* > 0.05) difference was observed in the percentage of muscle essential amino acid (EAA), non-essential amino acid (NEAA), and total amino acid (TAA), in spite of marked difference on the percentage of methionine (Met) and proline (Pro) among groups (Table 5). As presented in Table 6, the percentage of muscle saturated fatty acid (SFA) in T3 was significantly (*P* < 0.05) higher than T1 and T2, and lowest in T3, whereas, poly-unsaturated fatty acid (PUFA) percentage in T1 and T2 were significantly (*P* < 0.05) higher than T0, and lowest in T3.

**Table 4:** Muscle composition (%) of grass carp (means ± SD, *n* = 4).

**Groups**	**T0**	**T1**	**T2**	**T3**
Moisture	78.98 ± 0.71	80.22 ± 0.19	79.29 ± 0.13	78.95 ± 0.16
Ash	1.26 ± 0.02	1.19 ± 0.13	1.25 ± 0.04	1.27 ± 0.04
Protein	17.75 ± 0.03^c^	16.81 ± 0.07^a^	17.39 ± 0.11^b^	17.73 ± 0.13^c^
Lipid	2.12 ± 0.13^c^	1.78 ± 0.09^b^	1.62 ± 0.05^a^	1.72 ± 0.10^ab^
Glycogen (mg/g)	6.93 ± 0.45^a^	10.89 ± 1.10^bc^	11.49 ± 0.27^c^	9.77 ± 0.31^b^

*In the same line, different lower-case letters indicate significant differences (P < 0.05), values with no letter mean no significant difference (P > 0.05)*.

**Table 5:** Amino acid content in the muscle (% dry matter) of grass carp (means ± SD, *n* = 4).

	**T0**	**T1**	**T2**	**T3**
Lys	7.94 ± 0.28	8.01 ± 0.43	7.97 ± 0.44	7.71 ± 0.42
Met	2.03 ± 0.17^a^	2.17 ± 0.12^a^	2.48 ± 0.09^b^	2.13 ± 0.02^a^
Arg	5.14 ± 0.19	4.91 ± 0.49	5.22 ± 0.20	5.05 ± 0.27
His	3.36 ± 0.45	3.50 ± 0.47	3.34 ± 0.26	3.22 ± 0.41
Ile	4.29 ± 0.14	4.27 ± 0.18	4.26 ± 0.25	4.14 ± 0.21
Leu	7.16 ± 0.27	7.13 ± 0.32	7.09 ± 0.36	6.92 ± 0.32
Thr	3.65 ± 0.11	3.50 ± 0.20	3.64 ± 0.19	3.49 ± 0.17
Phe	4.47 ± 0.15	4.41 ± 0.20	4.38 ± 0.21	4.22 ± 0.25
Val	4.37 ± 0.11	4.35 ± 0.21	4.31 ± 0.24	4.18 ± 0.21
EAA^1^	42.40 ± 1.61	42.25 ± 2.32	42.69 ± 1.97	41.05 ± 2.23
Asp	8.68 ± 0.32	8.42 ± 0.40	8.56 ± 0.45	8.13 ± 0.37
Tyr	3.66 ± 0.12	3.73 ± 0.20	3.70 ± 0.20	3.56 ± 0.21
Ser	3.24 ± 0.10	3.19 ± 0.15	3.29 ± 0.15	3.08 ± 0.13
Glu	13.65 ± 0.47	13.25 ± 1.03	13.41 ± 0.30	13.15 ± 0.63
Gly	4.67 ± 0.27	4.51 ± 0.51	4.25 ± 0.42	4.16 ± 0.59
Ala	5.26 ± 0.19	5.04 ± 0.46	4.89 ± 0.29	4.92 ± 0.37
Cys	0.28 ± 0.23	0.21 ± 0.16	0.50 ± 0.01	0.35 ± 0.17
Pro	2.27 ± 0.12^ab^	2.35 ± 0.17^ab^	2.38 ± 0.14^b^	2.04 ± 0.22^a^
NEAA^2^	41.71 ± 0.78	40.70 ± 2.47	40.82 ± 1.43	39.40 ± 1.81
TAA^3^	84.11 ± 2.31	82.95 ± 4.73	83.51 ± 3.36	80.46 ± 3.88

*In the same line, different lower-case letters indicate significant differences (P < 0.05), values with no letter mean no significant difference (P > 0.05); 1. Essential amino acid; 2. Non-essential amino acid; 3. Total amino acid*.

**Table 6:** Fatty acid percentage in the muscle (% dry matter) of grass carp (means ± SD, *n* = 4).

	**T0**	**T1**	**T2**	**T3**
C14:0	0.22 ± 0.02	0.23 ± 0.01	0.22 ± 0.03	0.23 ± 0.01
C15:0	0.09 ± 0.00^a^	0.10 ± 0.01^b^	0.10 ± 0.01^ab^	
C16:0	5.44 ± 0.06^b^	5.53 ± 0.21^b^	5.41 ± 0.37^b^	4.93 ± 0.17^a^
C17:0	0.06 ± 0.00	0.06 ± 0.01	0.06 ± 0.01	
C18:0	1.64 ± 0.02^b^	1.65 ± 0.05^b^	1.62 ± 0.09^b^	1.45 ± 0.05^a^
C16:1n7	1.63 ± 0.14^ab^	1.67 ± 0.07^ab^	1.72 ± 0.18^b^	1.52 ± 0.06^a^
C17:1n8	0.08 ± 0.01^ab^	0.10 ± 0.01^b^	0.10 ± 0.01^b^	0.07 ± 0.01^a^
C18:1n5	0.90 ± 0.10^ab^	0.98 ± 0.07^b^	0.95 ± 0.09^ab^	0.82 ± 0.05^a^
C18:1n9	9.45 ± 0.91^ab^	10.50 ± 0.64^b^	10.45 ± 1.15^b^	8.41 ± 0.50^a^
C20:1n7	0.24 ± 0.03^ab^	0.27 ± 0.02^b^	0.28 ± 0.04^b^	0.21 ± 0.02^a^
C18:2n6	6.69 ± 0.49^b^	7.46 ± 0.46^bc^	7.61 ± 0.72^c^	4.99 ± 0.31^a^
C18:3n3	0.53 ± 0.03^b^	0.57 ± 0.03^b^	0.59 ± 0.06^b^	0.34 ± 0.04^a^
C18:3n6	0.10 ± 0.05	0.09 ± 0.04	0.15 ± 0.01	
C20:3n6	0.58 ± 0.03^b^	0.63 ± 0.05^b^	0.70 ± 0.05^c^	0.43 ± 0.03^a^
C20:3n9	0.29 ± 0.01^b^	0.36 ± 0.03^c^	0.34 ± 0.03^c^	0.25 ± 0.02^a^
C20:4n6	2.72 ± 0.15^b^	2.99 ± 0.27^b^	3.00 ± 0.15^b^	2.02 ± 0.17^a^
C20:5n3	0.50 ± 0.05^b^	0.60 ± 0.09^c^	0.50 ± 0.02^b^	0.36 ± 0.04^a^
C22:6n3	3.02 ± 0.11^b^	3.36 ± 0.34^c^	3.26 ± 0.13^bc^	2.12 ± 0.18^a^
ΣSFA^1^	7.44 ± 0.10^a^	7.57 ± 0.27^a^	7.41 ± 0.50^a^	6.60 ± 0.23^b^
ΣMUFA^2^	12.31 ± 1.08^b^	13.50 ± 0.66^c^	13.50 ± 1.77^c^	11.03 ± 0.68^a^
ΣPUFA^3^	14.42 ± 0.89^b^	16.05 ± 1.02^c^	16.14 ± 1.31^c^	10.52 ± 0.86^a^
n-3	4.05 ± 0.17^b^	4.53 ± 0.45^c^	4.35 ± 0.18^bc^	2.83 ± 0.27^a^
n-6	10.25 ± 0.73^b^	11.35 ± 0.60^bc^	11.65 ± 1.11^c^	7.58 ± 0.59^a^
n-3/n-6	0.40 ± 0.01	0.40 ± 0.02	0.37 ± 0.02	0.37 ± 0.01
DHA/EPA	6.06 ± 0.36^ab^	5.66 ± 0.54^a^	6.54 ± 0.43^b^	5.83 ± 0.36^a^
EPA/ARA	0.18 ± 0.01^ab^	0.20 ± 0.02^b^	0.17 ± 0.01^a^	0.18 ± 0.01^ab^

*In the same line, different lower-case letters indicate significant differences (P < 0.05), values with no letter mean no significant difference (P > 0.05); 1. saturated fatty acid; 2. mono-unsaturated fatty acids; 3. poly-unsaturated fatty acids*.

### Digestive Enzyme Activities

Intestinal activities of trypsin and α-amylase significantly (*P* < 0.05) increased with dietary tannin level, while the lipase activity of T0 and T1 was significantly (*P* < 0.05) higher than T2 and T3 (Table 7).

**Table 7:** Digestive enzyme activities (Umg^−1^ protein) in intestine of grass carp (means ± SD, *n* = 4).

	**T0**	**T1**	**T2**	**T3**
Trypsin	2503.95 ± 49.82^a^	2797.27 ± 29.02^b^	3078.11 ± 154.12^c^	3169.59 ± 88.02^c^
α-Amylase	17.10 ± 0.12^a^	18.01 ± 0.59^b^	18.99 ± 0.15^c^	19.78 ± 0.09^d^
Lipase	4.71 ± 1.05^b^	4.97 ± 0.76^b^	2.10 ± 0.10^a^	2.28 ± 0.41^a^

*In the same line, different lower-case letters indicate significant differences (P < 0.05)*.

### Hepatopancreas and Serum Biochemical Parameters

The hepatopancreas and serum biochemical parameters are presented in Tables 8, 9. In hepatopancreas, the activity of AST in T0 and T1 was significantly (*P* < 0.05) higher than T2 and T3, and the activity of ALT of T0 was significantly (*P* < 0.05) higher than T1, while lowest in T2 and T3 (*P* < 0.05). The hepatic glycogen content significantly (*P* < 0.05) increased with dietary tannin level and decreased at T3 (Table 8).

**Table 8:** Hepatopancreas parameters of grass carp (means ± SD, *n* = 4).

	**T0**	**T1**	**T2**	**T3**
AST (U/mg)	30.57 ± 1.99^b^	29.36 ± 0.99^b^	24.12 ± 1.84^a^	24.31 ± 0.64^a^
ALT (U/mg)	122.66 ± 1.78^c^	112.85 ± 6.24^b^	93.92 ± 2.34^a^	93.91 ± 1.35^a^
Hepatic glycogen (mg/g)	5.08 ± 0.07^a^	6.56 ± 0.14^c^	7.82 ± 0.18^d^	6.19 ± 0.09^b^

*In the same line, different lower-case letters indicate significant differences (P < 0.05). ALT, alanine aminotransferase; AST, aspartate aminotransferase*.

**Table 9:** Serum parameters of grass carp (means ± SD, *n* = 4).

	**T0**	**T1**	**T2**	**T3**
Total protein (gprot/L)	44.38 ± 0.65^b^	45.64 ± 0.35^b^	53.01 ± 1.55^c^	41.44 ± 1.41^a^
Albumin (g/L)	17.53 ± 0.16^a^	24.04 ± 1.06^b^	16.69 ± 1.13^a^	16.78 ± 0.42^a^
Globulin (g/L)	26.85 ± 0.49^b^	21.61 ± 0.81^a^	36.32 ± 2.64^c^	24.66 ± 1.34^b^
Urea nitrogen (mmol/L)	6.18 ± 0.15^d^	2.83 ± 0.06^b^	3.40 ± 0.10^c^	2.23 ± 0.07^a^
Triglyceride (mmol/L)	2.68 ± 0.06^b^	3.18 ± 0.07^c^	3.24 ± 0.09^c^	2.39 ± 0.08^a^
Total cholesterol (mmol/L)	2.68 ± 0.02^a^	3.73 ± 0.08^b^	5.04 ± 0.20^c^	2.59 ± 0.04^a^
LDL-C (mmol/L)	1.34 ± 0.07^c^	1.51 ± 0.09^d^	0.82 ± 0.03^b^	0.54 ± 0.06^a^
HDL-C (mmol/L)	7.43 ± 0.18^c^	5.70 ± 0.19^b^	5.17 ± 0.44^bc^	4.95 ± 0.29^a^
HDL-C/LDL-C ratio	5.55 ± 0.36^b^	3.80 ± 0.33^a^	6.33 ± 0.75^b^	9.24 ± 1.47^c^

*In the same line, different lower-case letters indicate significant differences (P < 0.05). LDL-C, Low density lipoprotein cholesterol; HDL-C, High density lipoprotein cholesterol*.

In serum, the TP content of T2 was significantly (*P* < 0.05) higher than T0 and T1, and lowest in T3 (Table 9). While ALB content of T1 was significantly (*P* < 0.05) higher than other groups, the globulin of T2 was significantly (*P* < 0.05) higher than T0 and T3, and lowest in T1. The UN content of T0 was significantly (*P* < 0.05) higher than T2 and T1, and lowest in T3. The triglyceride and TC significantly (*P* < 0.05) increased with dietary tannin level and then decreased in T3. The LDL-C content of T1 was significantly (*P* < 0.05) higher than T0, T0 is significantly (*P* < 0.05) higher than T2 and lowest in T3. The HDL-C content significantly (*P* < 0.05) decreased with dietary tannin level.

### Reactions of mRNA Gene Expressions to Tannin Levels in Intestine and Hepatopancreas

**Figure 1:**
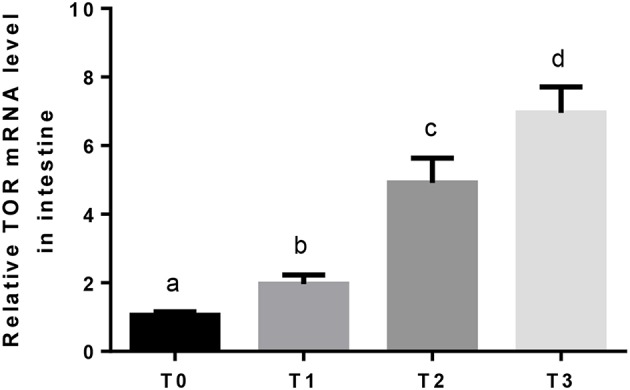
The relative expression of TOR mRNA in the intestine. The letters indicate the results of multiple range test, the different letters indicated significant differences (*P* < 0.05).

The relative expression of TOR mRNA in intestine was significantly (*P* < 0.05) upregulated as dietary tannin increased (Figure 1).

**Figure 2:**
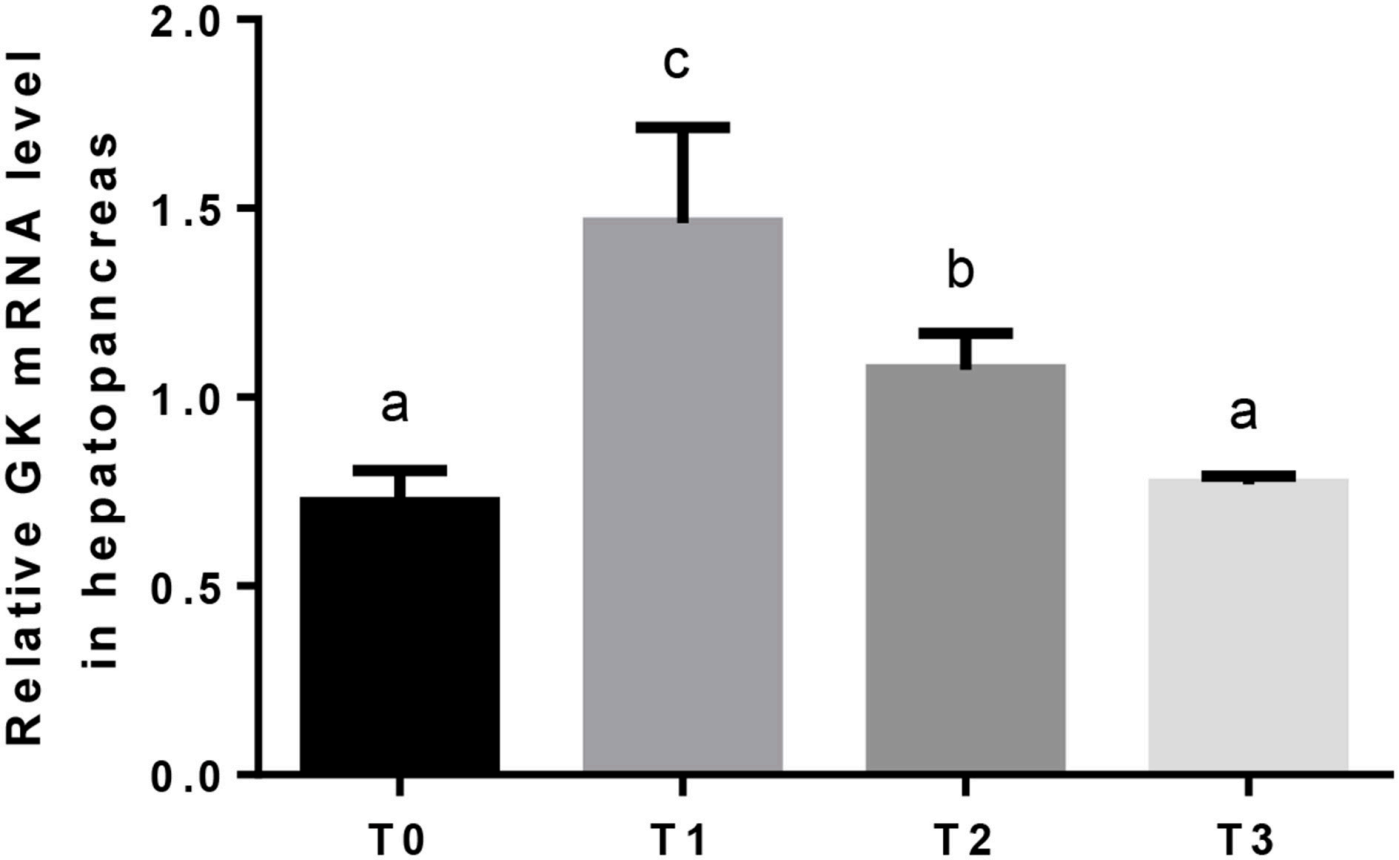
The relative expression of GK mRNA in the hepatopancreas. The letters indicate the results of multiple range test, the different letters indicated significant differences (*P* < 0.05).

**Figure 3:**
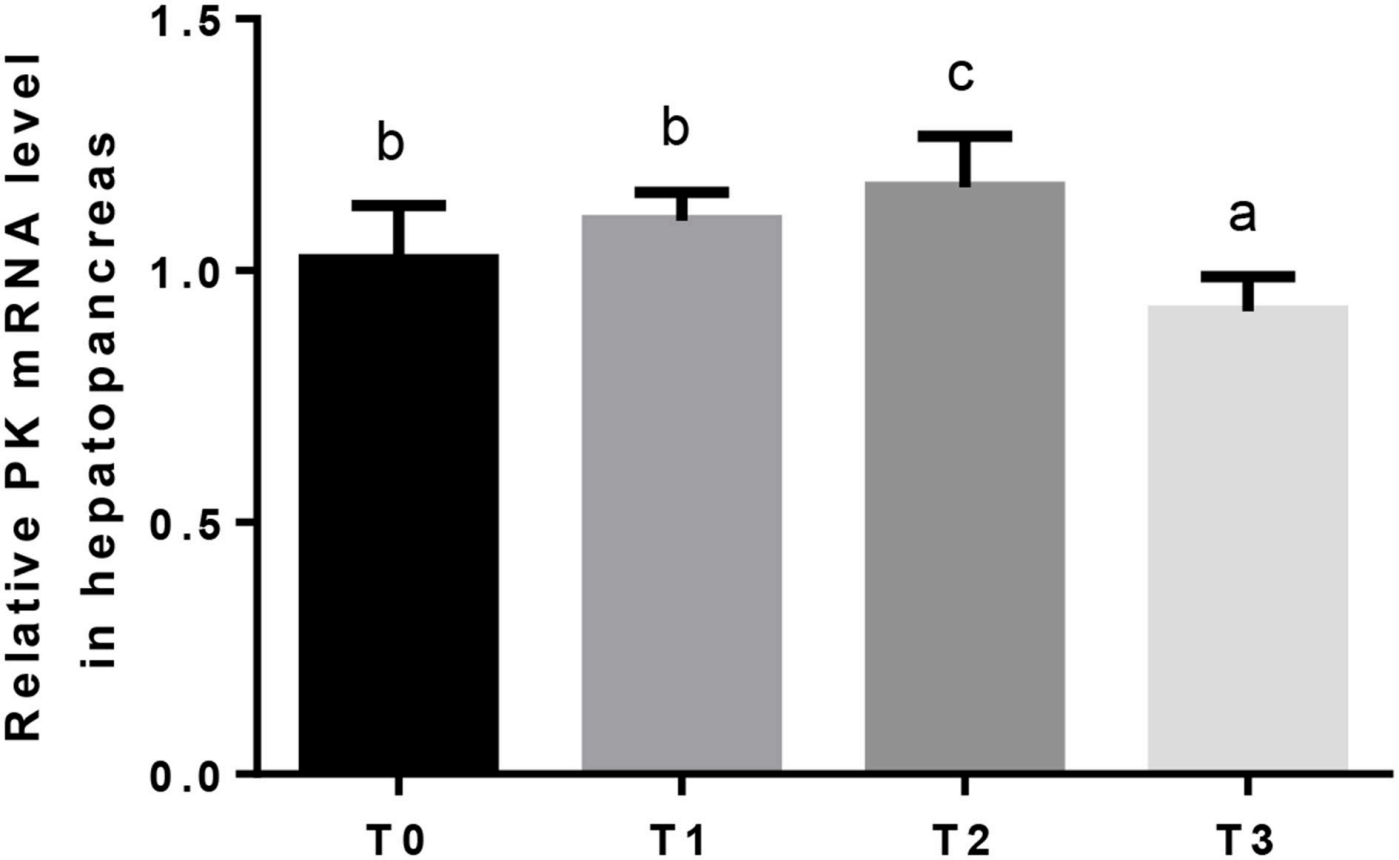
The relative expression of PK mRNA in the hepatopancreas. The letters indicate the results of multiple range test, the different letters indicated significant differences (*P* < 0.05).

Hepatic GK mRNA in T1 was significantly (*P* < 0.05) higher than T2, and lowest in T0 and T3 (Figure 2). The relative expression of hepatic PK mRNA in T2 was significantly (*P* < 0.05) higher than T0 and T1, and lowest in T3 (Figure 3).

**Figure 4:**
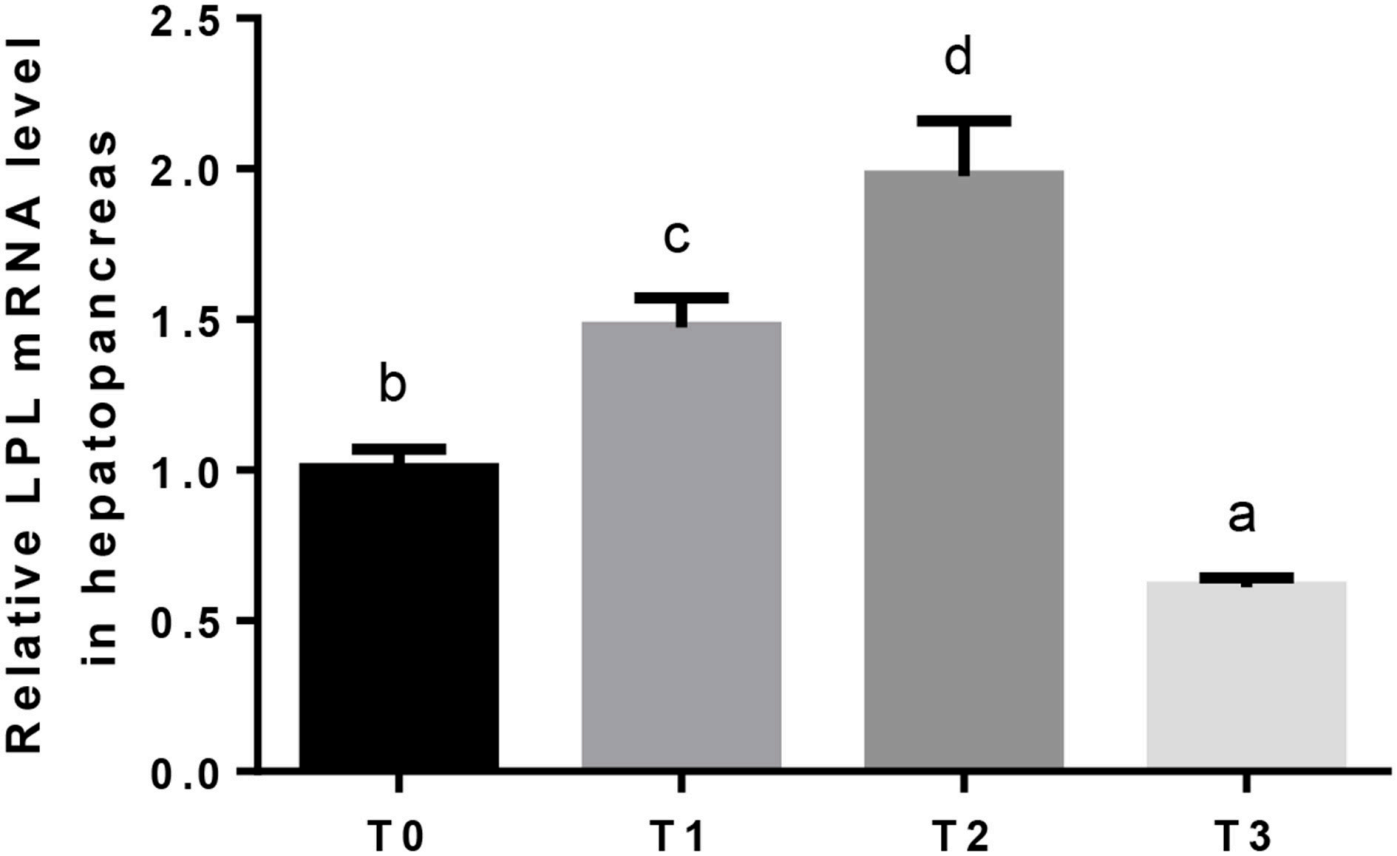
The relative expression of LPL mRNA in the hepatopancreas. The letters indicate the results of multiple range test, the different letters indicated significant differences (*P* < 0.05).

**Figure 5:**
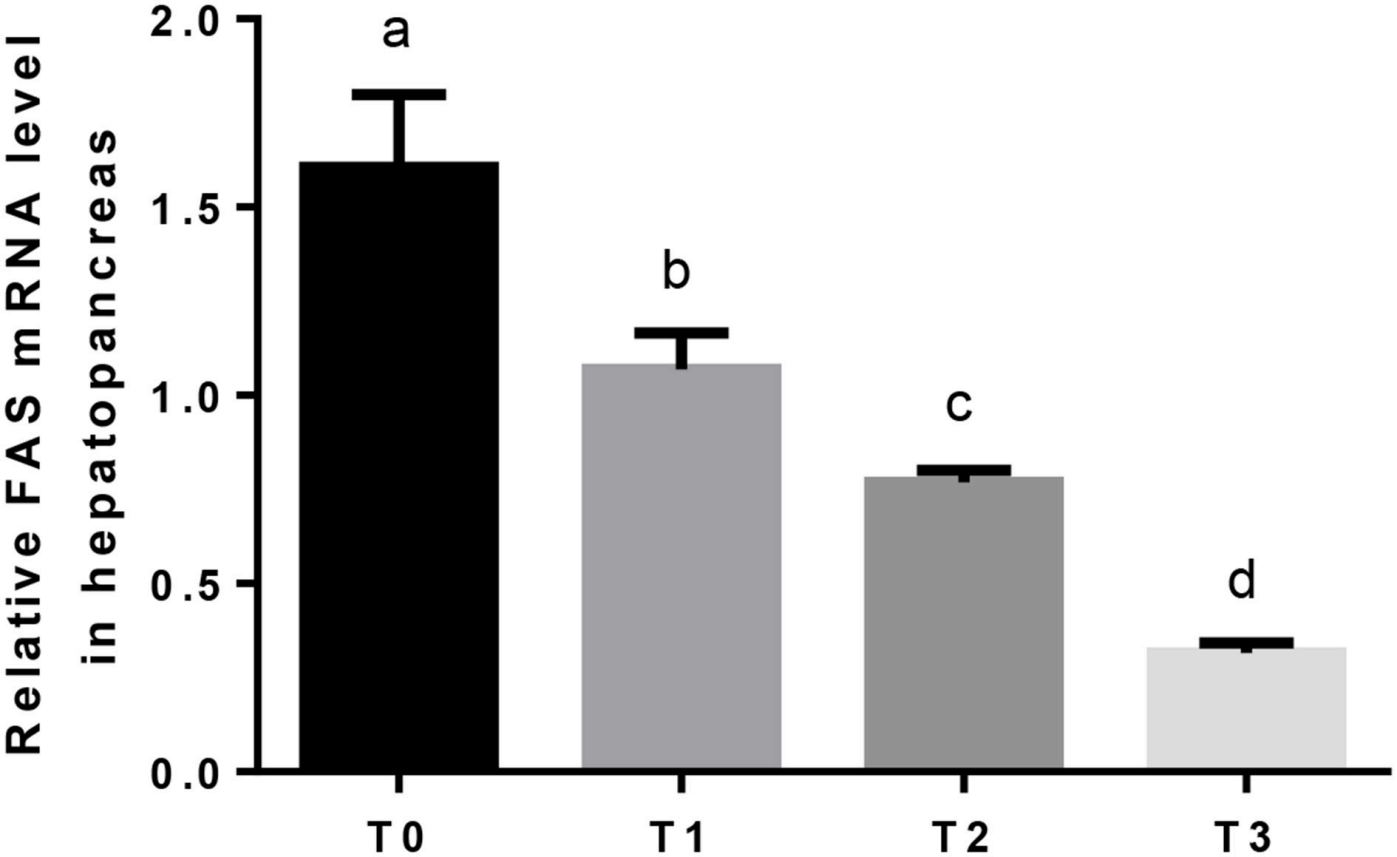
The relative expression of FAS mRNA in the hepatopancreas. The letters indicate the results of multiple range test, the different letters indicated significant differences (*P* < 0.05).

**Figure 6:**
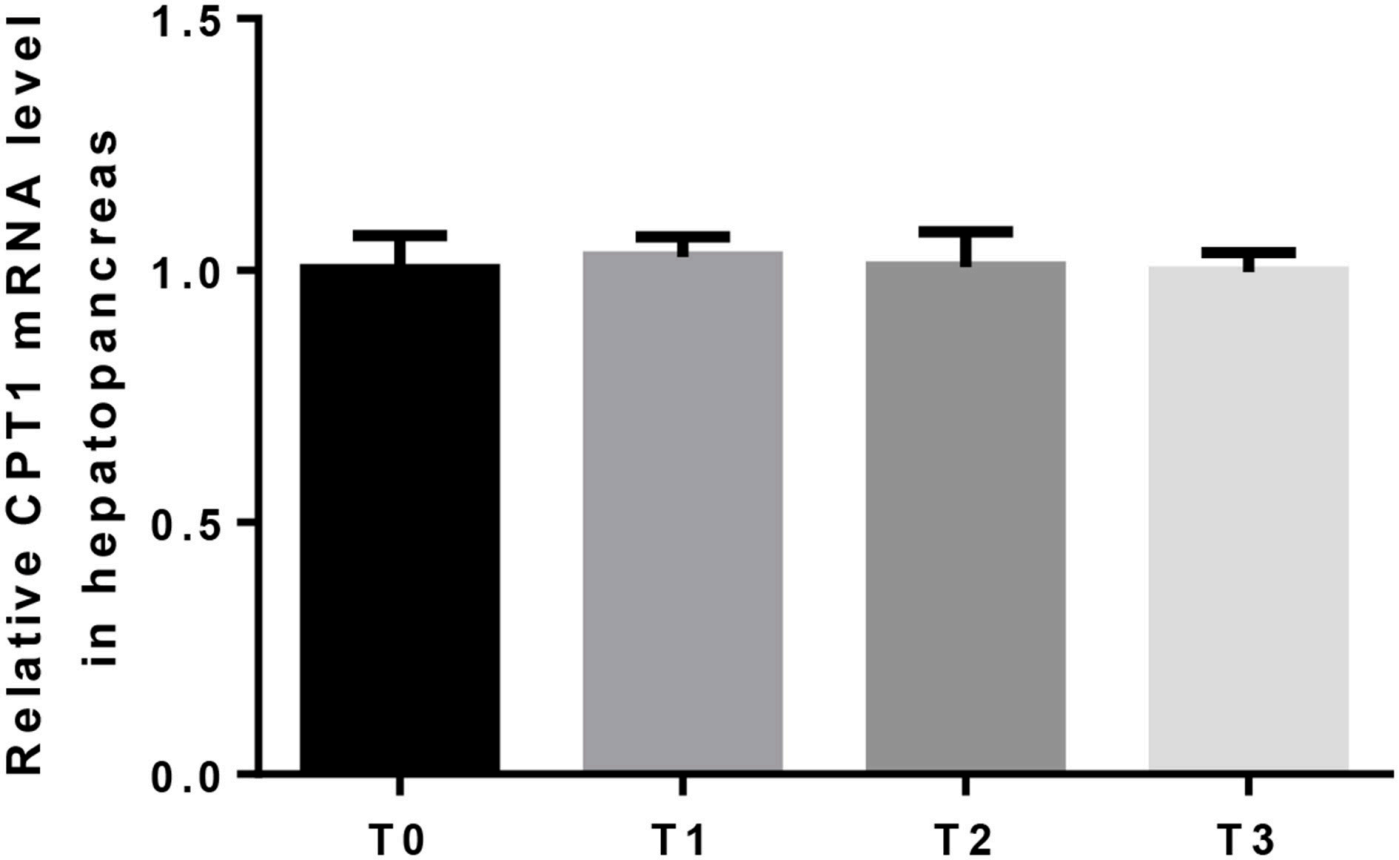
The relative expression of CPT1 mRNA in the hepatopancreas. The letters indicate the results of multiple range test, no letter indicated no significant differences (*P* > 0.05).

The mRNA expression of hepatic LPL was significantly (*P* < 0.05) upregulated as tannin level and downregulated in T3 (Figure 4). The relative expression of FAS mRNA downregulated significantly (*P* < 0.05) as tannin level (Figure 5). No significant (*P* > 0.05) difference was observed in the relative expression of hepatic CPT1 mRNA (Figure 6).

## Discussion

### The Effect of Tannin on Growth Performance

In order to protect global fish stocks and reduce costs, numerous studies on replacing fishmeal by other protein sources have been demonstrated. Tannin, as one of the ANFs, is widely present in plant products, such as rapeseed meal, seaweed, hazelnut seed, soybean, wheat, sunflower, field pea, chickpea, sorghum, and microalgal biomass (37–41). Therefore, the tolerable threshold and effect of tannin on fish should be investigated.

In the present study, no apparent negative effect was observed on growth performance, but the FI and FCR increased. Similar results were reported in the study of Hamilton (*Labeo rohita*) fingerlings when 2% dietary hydrolysable tannin was added in their diet (27). However, when 2.5% dietary hydrolysable tannin was added in the diet of Nile Tilapia (*Oreochromis niloticus*), the weight gain decreased and the FCR increased (20). Similar results were obtained when 3% dietary tannin was added in the diet of European seabass (*Dicentrarchus labrax* L) (42). It has been indicated that mammalian herbivores have an innate defensive mechanism against dietary polyphenolics; the existence of proline rich proteins in their saliva would bind with tannin and protect them against polyphenolics (43). It is reasonable to assume that, the tolerable threshold of dietary tannin was connected with feeding habits, and herbivorous fish could tolerate higher level of tannin without influencing the growth compare to omnivorous and carnivorous fish. Furthermore, different energy sources may be importance as well, where studies have evaluated that, carnivorous fish rely heavily on protein and lipid as energy sources, while omnivorous and herbivorous fish have a greater proclivity for utilizing plant feedstuffs and carbohydrate for energy (44). Consequently, grass carp may have strong adaptability to tannin and establish a special regulatory mechanism of growth.

### The Effect of Tannin on Protein Metabolism

Protein is a major energy source for aquatic organisms, and the growth of animal is connected with nutrient deposition especially protein deposition for fish (45).

The deposition of protein is related to the balance of absorption and biosynthesis. McCurdy and March (46) reported that, tannins bind with dietary proteins and digestive enzyme, and reduce the digestibility of proteins as well as diet palatability. This is in agreement with the results from Nile tilapia (20) and European seabass (42) that, the digestibility of protein showed an inverse relationship with dietary tannin levels. However, in the present study, the activity of trypsin increased with dietary tannin levels. Tannins are known to interfere with digestive enzymes, and thereby, in order to increase digestibility, more trypsin needs to be secreted. Therefore, we put forward the hypothesis that, with the increased activity of trypsin, the digestibility of protein does not always increase. This speculation is supported by the increasing expression of TOR mRNA in intestine. TOR pathway is closely linked with protein synthesis, which could modulate protein translation, stimulate protein synthesis and regulate cellular growth, proliferation and metabolism (47, 48). In the present study, the increasing expression of TOR mRNA may promote the synthesis of digestive enzymes.

Serum TP, ALB and UN are important indicators in protein metabolism, of which UN is the main end products of protein metabolism. Low TP and ALB content indicate poor nutrition and protein metabolic status in aquatic organisms. Globulin is a crucial indictor in fish immunity, which would increase when fish is injured (49). In the present study, the TP content in serum decreased in T3, and ALB decreased in T2, which indicated that, excess dietary tannin impacted the metabolism of protein. In addition, the content of serum UN decreased and globulin increased in T2, which indicated that, the catabolism of amino acids decreased and hepatopancreas may be damaged to a certain extent (50). Pervious study demonstrated that, tannin partially inhibit the antioxidant enzymes and induct the damage of liver (51). In a study of carp (*Cyprinus carpio* L), when 10 mg/kg body tannin were injected, the toxicity for fish due to oxidative stress induction and antioxidant enzyme inhibition were observed (52).

AST and ALT are the main aminotransferase in hepatopancreas. The aminotransferases catabolize amino acids which are absorbed in intestine and transfer amino groups to alpha-keto acids (53). In the present study, the activities of hepatic AST and ALT reduced with dietary tannin levels, which indicated that, the biosynthesis of protein was injured. Deficient digestible amino acids derived by tannin may be an important reason and the hepatopancreas may present a tendency to be injured (53), as hydrolysable tannins are readily hydrolyzed in the intestine to small phenolic compounds that are absorbed and may cause toxicity to hepatopancreas (52, 54). In mice, a single subcutaneous injection of tannin at 700 mg/kg body weight caused a significant break down of polyribosomes in liver and inhibited the incorporation of amino acids into hepatic proteins (10). When rat and chick were fed with diet containing 1.6% tannin extract from faba beans, hydropic degeneration of hepatocytes were observed (55) and a similar result was also presented in goat (56).

The above results manifested that, when 1.25% dietary tannin was fed to grass carp, the digestion, biosynthesis, and catabolism of protein were impaired, the antinutritional function and toxicity of tannin may be responsible for these.

### The Effect of Tannin on Lipid Metabolism

Lipid and digestible carbohydrate are the major non-protein energy sources in fish diets, but as a typical herbivorous fish like grass carp, excessive lipid accumulation occurs in practical culture (57, 58). Excessive lipid accumulation in fish body and tissue affects the quality in nutritional value and injure the health of fish by lipid oxidation and peroxidation (59, 60). In the present study, dietary tannin decreased muscle lipid content. Similar result was reported in European seabass and Nile Tilapia where dietary tannin was supplemented at 2 and 1.5%, respectively (20, 42). The reduced muscle lipid was probably due to lower digestibility induced by low activity of lipase (61). In the present study, the lipase activity in intestine decreased in T2 and T3. Similar result was reported in rat fed diet contained 2.5% tannin (21). Previous study reported that, because of the structure of lipase, the binding strength between the tannin and lipases was large and hard to modify *in vivo*, and this partly account for the low muscle lipid content and lipase activity (21, 62). The increases in muscle PUFAs percentages in T1 and T2, may be suggested to be influenced by the anti-oxidative properties of tannin (63, 64).

Hepatopancreas is the main crossing organ for exogenous and endogenous transport of lipids. FAS is the key regulatory enzymes in fatty acid synthesis (65, 66). In the present study, the relative expression of hepatic FAS downregulated with dietary tannin levels, indicating that the biosynthesis of lipid was influenced.

LDL-C transports cholesterol from liver to peripheral tissues, while HDL-C mediates the reverse transport of cholesterol from peripheral tissues to the liver, and HDL-C/LDL-C ratio is a marker of cholesterol transport (67, 68). It has been shown that tannin could reduce serum cholesterol levels (69). In a study on rats injected 30 mg/week hydrolysable tannin, serum cholesterol and triglycerides reduced, and lipogenesis was suppressed by insulin (70, 71). This may because that tannin could bind with, increase fecal excretion of bile acids and acceleration of cholesterol catabolism to a level that is greater than that of cholesterol synthesis in the liver (72–74). In the present study, however, the total cholesterol (TC) content in serum increased with the dietary tannin levels and decreased in T3, whereas HDL-C decreased, and LDL-C increased in T1 and then decreased, and the HDL-C/LDL-C ratio decreased in T1 and then increased. These indicate that, 1.25% dietary hydrolysable tannin would improve cholesterol transport in grass carp.

In general, fat accumulation results from the balance between absorbed dietary lipid, lipogenesis and lipolysis. In fish, the metabolic pathways of lipid biosynthesis and oxidation are essentially the same as those observed in mammals (75). Through oxidation, the fatty acids can be used as energy source, or stored and deposited in adipose tissues (76). LPL is considered to be a key enzyme in the lipid deposition and metabolism, which hydrolyzes triacylglycerols present in plasma lipoproteins and supplies free fatty acids for storage in adipose tissue, or for oxidation in other tissues (77–79). In the present study, the relative expression of hepatic LPL mRNA upregulated and subsequently downregulated in T3, and the serum triglyceride showed similar tendency with LPL. Carnitine palmitoyltransferase I (CPT1) is considered to be the main regulatory enzyme in long-chain fatty acid oxidation which catalyzes the conversion of fatty acid-CoAs into fatty acid-carnitines for entry into the mitochondrial matrix (80). The relative expression of CPT1mRNA did not show significant differences. These results suggest that, fish reduced lipid deposition when fed diet contained hydrolysable tannin, and the oxidation of fatty acid was not injured. 1.25% dietary tannin seems the best level to decrease the deposition of lipid and increased cholesterol transport.

### The Effect of Tannin on Carbohydrate Metabolism

Compared to dietary lipid, carbohydrate is relatively cheaper and a readily available source of energy to herbivorous fish (81). Higher amylase activity in intestine and metabolic enzyme activity are important reasons (82). After carbohydrate is digested, the glycogen is mainly deposited in muscle and liver. Liver glycogen content indicates accumulation in the liver as both lipid and glycogen after conversion (83), and muscle glycogen serve as an energy reserve for that particular organ (84). In the present study, the activity of amylase increased with dietary tannin levels, and the glycogen content in hepatopancreas and muscle increased and then decreased in T3. These indicate that, dietary tannin improved the digestion and accumulation of carbohydrate.

Glycolysis is the major route of glucose metabolism in fish as in other animals (85, 86). Hepatic GK and PK are important enzymes that play crucial roles in glycolysis regulation. GK removes glucose from the blood after feeding and partakes in phosphorylation of glucose as the first step of glucose utilization, and PK catalyzes the last step of glycolysis, the conversion of phosphoenolpyruvate to pyruvate (87, 88). In the present study, when dietary tannin was supplemented, the relative expression of GK in hepatopancreas upregulated, and the relative expression of PK upregulated in T2 and then downregulated. The elevated GK and PK transcript induce an increase in glucose uptake from circulation into hepatopancreas, and increase glucose storage in hepatopancreas for subsequent energy supply. Based on above results, it was considered that, 0.75 and 1.25% dietary tannin could improve the digestion and glycolysis of carbohydrate, and these may spare the consumption of protein and lipid for energy (89, 90).

In conclusion, grass carp could tolerate 1.75% dietary hydrolysable tannin without influencing growth. However, 1.25% dietary tannin impaired the digestion and metabolism of protein. But the utilization of carbohydrate was promoted, the digestion and biosynthesis of lipid decreased, and lipolysis increased to decrease the deposition of lipid and increase cholesterol transport. Furthermore, the present study provides a theoretical reference to the use of tannin-rich plant sources in aquatic feed. From this perspective of tannin, dietary rapeseed meal in grass carp should not exceed 50%, and dietary broad bean should not exceed 75%.

## Data Availability Statement

All datasets generated for this study are included in the article/supplementary material.

## Ethics Statement

All procedures were carried out according to national and institutional regulations on the care and use of experimental animals. The experimental handling and treatment of experimental fish were conducted in accordance with the regulations made by the Institutional Animal Care and Use Committee (IACUC), Shanghai Ocean University (SHOU), and this study was approved by the IACUC of SHOU, Shanghai, China.

## Author Contributions

Six authors are justifiably credited with authorship. XH, JY, and PC conceived the study and designed the experiments. JY, GZ, and ZH contributed to performing the experiment. JY and PC did the data analysis and wrote the paper. XH and AA revised the manuscript.

### Conflict of Interest

The authors declare that the research was conducted in the absence of any commercial or financial relationships that could be construed as a potential conflict of interest.
